# Acute Coronary Syndrome in a Male with Elevated Anti-Cyclic Citrullinated Peptide and no Evidence of Longstanding Rheumatoid Arthritis

**DOI:** 10.31138/mjr.31.3.362

**Published:** 2020-09-30

**Authors:** Georges El Hasbani, Ziyad Ghazzal, Habib Dakik, Lamiaa Hamie, Imad Uthman

**Affiliations:** Department of Internal Medicine, American University of Beirut, Lebanon

**Keywords:** Acute coronary syndrome, rheumatoid arthritis, biomarkers, risk factors

## Abstract

A 55-year-old male, previously known to be healthy, presented to the emergency department with a 30-minute history of chest pain radiating to the upper extremities. Vital signs were within normal limits. Four days prior to this presentation, the patient presented for acute onset of polyarthritis and morning stiffness. Significantly elevated titres of anti-cyclic citrullinated peptides (anti-CCP) were found. In the emergency department, electrocardiography showed ST segment elevations in leads V1 to V5 and aVL. Cardiac enzymes were elevated. The patient underwent cardiac catheterization. A coronary angiography revealed an ectatic proximal left anterior descending (LAD) coronary artery with critical (90–99%) stenosis at the mid segment. A drug-eluting stent was successfully inserted in the LAD without any residual stenosis. Although it is known that anti-CCP positivity is a key element in the pathogenesis of atherosclerosis in RA patients, this case report adds to the existing body of literature which demonstrates that anti-CCP positivity is an independent risk factor for development of cardiovascular events.

## INTRODUCTION

It is well established that patients with rheumatoid arthritis (RA) have an increased risk of cardiovascular disease in general, and ischemic heart disease (IHD) in particular.^1^ The clinical outcome and radiological severity of RA have been linked with specific biomarkers such as the antibodies against citrullinated proteins and cyclic citrullinated peptides (anti-CCP).^2^ Since citrullinated proteins are prevalent within atherosclerotic plaques, anti-CCP were associated with atherosclerotic burden in patients with RA.^3^ However, the existing data in the literature between non-RA anti-CCP positivity and cardiovascular disease (CVD) risk is still unclear. Herein, we discuss the case of a previously healthy 55-year-old man who had a myocardial infarction after a few days of developing acute polyarthritis in his metacarpophalangeal (MCP) joints and highly elevated titres of anti-CCP. The patient did not meet the criteria of RA thereafter.

## CASE DESCRIPTION

**Figure 1.**
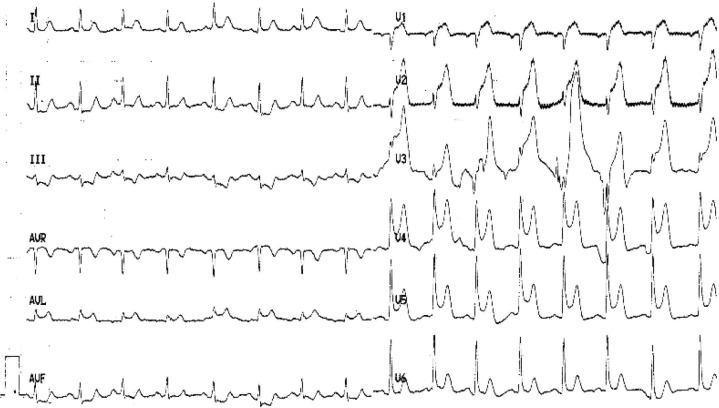
ECG shows ST segment elevation in leads aVL and V1 to V5.

A 55-year-old male, previously known to be healthy, presented to the emergency department with a 30-minute history of oppressive chest pain radiating to the upper extremities. Upon arrival, his vital signs were as follows: Blood pressure, 112/76 mmHg; pulse rate, 84 beats per minute; and peripheral oxygen saturation on room air, 96%. The patient did not have any family history of coronary artery disease or any other risk factors such as smoking or alcohol abuse. The physical examination was completely normal with no S3 or S4 sounds documented. Electrocardiography (ECG) before securing venous route showed ST segment elevations in leads V1 to V5 and aVL (**Figure 1**).

The patient underwent an immediate cardiac catheterization which revealed an ectatic proximal left anterior descending (LAD) coronary artery with critical (90–99%) stenosis at the mid segment, mild ostial disease (20%) in the circumflex artery, and mild ectasia in the right coronary artery (**Figure 2A**). A drug-eluting stent was successfully inserted in the LAD without any residual stenosis (**Figure 2B**). Left ventriculogram showed an ejection fraction of 60–64% with a hypokinetic anterior myocardial wall. The results of a biochemical blood analysis performed upon presentation were significant for elevated troponin T levels of 11294 pg/mL and creatine kinase myocardial band (CK-MB) of 876 IU/L. His haemoglobin, white blood count, platelet count, liver function tests, thyroid function tests, lipid levels, fasting blood sugar, prothrombin time, activated partial thromboplastin time, fibrinogen, and D-dimer were all within normal limits. Patient showed prompt recovery and had a good follow-up course.

**Figure 2A.**
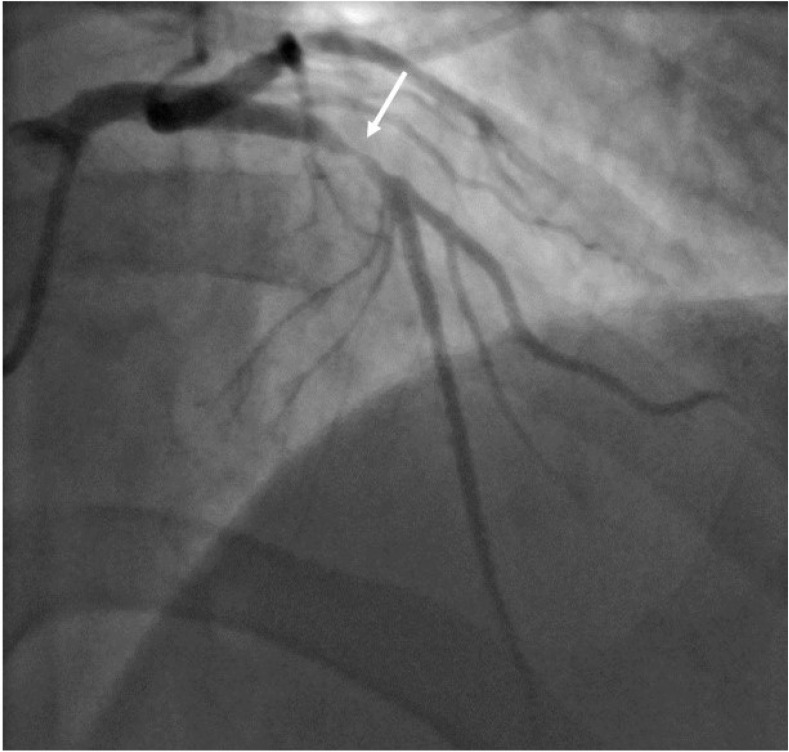
Initial coronary angiogram showing severe stenosis (95%) in the proximal LAD artery (Arrow).

**Figure 2B.**
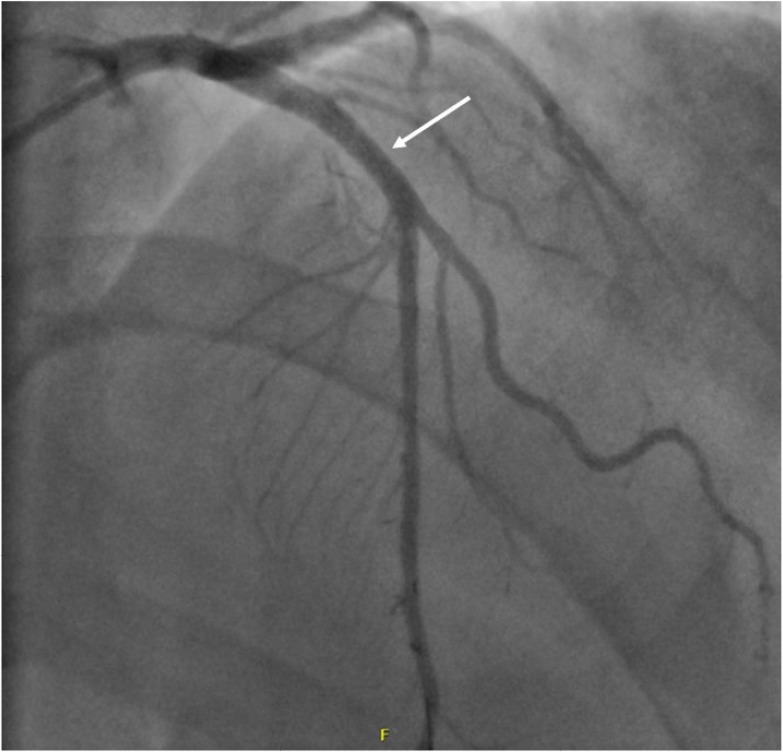
Coronary angiogram post successful coronary angioplasty and stenting (Arrow).

Four days prior to the event of myocardial infarction, the patient was complaining of 3-day history of acute polyarthritis and morning stiffness in the MCP joints of both hands that improves within 2 hours. No pain or other systemic symptoms were reported. Work-up to rule out reactive arthritis or new onset inflammatory arthritis showed a strongly positive anti-CCP of 500 u/ml (Normal <20), negative rheumatoid factor (RF), negative Brucella serology, and an elevated C-Reactive Protein (CRP) of 46 mg/dL. The patient did not report taking any non-steroidal anti-inflammatory drugs (NSAIDs) or pain-killer pending the blood tests and did not initiate any disease-modifying anti-rheumatic drugs (DMARDs) except after recovery of the acute myocardial infarction (AMI) event. The patient did not meet RA diagnostic criteria after a 6-month period of follow-up.

## DISCUSSION

In RA patients, anti-CCP antibodies are potentially important surrogate markers for diagnosis and prognosis. These antibodies act as an independent predictor of radiological damage and disease progression.^4^ Arnab and colleagues associated anti-CCP positivity in established RA with subclinical CVD in the form of intimal medial thickness.^5^

Some studies stressed on the role of anti-CCP in the development of AMI secondary to inflammation in non-RA patients as our patient who did not meet the American College of Rheumatology (ACR) criteria for RA. The first evidence for an association between any anti-CCP antibodies and CAD in the absence of coexistent autoimmune rheumatic disease came by Cambridge and colleagues.^6^ Fantus et al.^7,8^ noted in their conference abstract, as well as in subsequent chart review, that the risk of CVD is increased with extent of anti-CCP elevation although the results were not statistically significant. Recently, Hermans and colleagues^9^ found in their cross-sectional study that approximately 11% of non-RA patients with established CAD are anti-CCP positive. All studies which discussed the increased risk of CV events in anti-CCP non-RA patients or early RA patients are summarized in **Table 1**.

**Table 1.** Studies which showed a significant association between elevated anti-CCP titers and risk of CAD development.

**Study**	**Type of study**	**Results**
Cambridge et al. (6) (2013)	Case control	10.4% of cases were anti-CCP positive compared to 3.8% of controls. The Odds ratio (OR) (95% Confidence Interval) remained significant after adjustment for classical risk factors including smoking and C-reactive protein. OR 3.26 (1.36–7.80), p = 0.008 after adjustment, and 4.23 (1.22–14.61) p = 0.02 before adjustment.
Fantus et al. (7) (2016)	Retrospective chart review	A total of 1721 records were included, with 399 RA patients and 1322 non-RA. The association between elevated anti-CCP levels and higher prevalence of CAD and cerebrovascular disease was significant in all patients and RA patients, but not in non-RA.
Hermans et al. (9) (2017)	Cross-sectional	In total, 29 (11%) of 275 AMI included patients were anti-CCP positive. When comparing anti-CCP positive patients to anti-CCP negative patients, an increased cumulative cardiac mortality was observed. After correction for other associated factors, anti-CCP positivity was associated with long-term mortality (Hazard Ratio 3.1 [CI 1.4–7.1], p-value = 0.01) and long-term combined endpoint of re-infarction and death (Hazard Ratio 2.4 [1.2–4.6], p-value = 0.01).

On the other hand, Innala and colleagues^10^ followed prospectively early RA patients for the developments of CV events. No association was found between the presence of RF or anti-CCP and future CV events in early RA patients.

## CONCLUSION

We present this case as additional evidence on the association between anti-CCP positivity and risk of CAD.Since the evidence presented in the literature is still controversial, future research is required to provide more insight into the development of citrulline induced auto-immunity in patients with CAD without RA, and into the mechanisms of atherosclerosis mediated by autoantibodies.
